# Extent and Mitigation of Financial Toxicity of Immune Checkpoint Inhibitors for the Treatment of Advanced Hepatocellular Carcinoma

**DOI:** 10.1177/10732748251399259

**Published:** 2025-11-26

**Authors:** Nabil E. Omar, Hazem Elewa, Kareem A. El-Fass, Dario Trapani, Said Dermime, Daoud Al-Badriyeh, Dina Abushanab, Anas Hamad

**Affiliations:** 1 Pharmacy Department, National Centre for Cancer Care and Research, Hamad Medical Corporation, Doha, Qatar; 2 Clinical and Population Health Research Program, College of Pharmacy, QU Health, Qatar University, Doha, Qatar; 3 College of Pharmacy, QU Health, Qatar University, Doha, Qatar; 4 724362 Syreon Middle East, Alexanderia, Egypt; 5 European Institute of Oncology, IRCCS, Department of Oncology and Hemato-oncology (DIPO), University of Milan, Milan, Italy; 6 Translational Cancer Research Facility, National Center for Cancer Care and Research, Hamad Medical Corporation, Doha, Qatar; 7 College of Health Sciences, QU Health, Qatar University, Doha, Qatar; 8 Drug Information Department, Hamad Medical Corporation, Doha, Qatar

**Keywords:** hepatocellular carcinoma, immune checkpoint inhibitors, low- and middle-income countries, financial toxicity

## Abstract

Advanced hepatocellular carcinoma (HCC) is a serious condition associated with significant morbidity and mortality. Over the past few decades, however, this has drastically changed, primarily attributed to the development of new treatments, including tyrosine-kinase inhibitors (TKIs), vascular endothelial growth factor (VEGF) inhibitors, and immune checkpoint inhibitors (ICIs). This study aims to highlight the financial toxicity (FT) of advanced HCC treatments, across diverse healthcare systems and particularly in low- and middle-income countries (LMICs). We also aim to explore potential solutions to improve access to life-saving therapies such as ICIs. We conducted an exploratory targeted review of the current literature focusing on therapeutic advancements, their accessibility, and innovative strategies to overcome FT. The review particularly addresses barriers faced in LMICs and examines initiatives that could optimize ICI availability. Findings suggest that FT significantly limits access to ICIs globally, with heightened impact in resource-limited settings and increasingly recognized in high-income countries as well. This challenge is particularly severe in LMICs, where the high incidence of HCC is compounded by the unaffordability of effective treatments, creating a significant barrier to care. Key strategies to mitigate this include cost-saving measures such as dose rounding, vial sharing, lower dosage regimens, extended dosing intervals, and shorter treatment durations. Evidence from emerging studies, predominantly in non-HCC malignancies, suggests these measures may be tolerated without unexpected safety signals. However, HCC-specific prospective data remain limited, and these approaches are entirely off-label requiring further research. Addressing financial barriers to ICI access is essential for improving outcomes in advanced HCC, globally and particularly in LMICs. Global adoption of cost-saving strategies could enhance equitable access to innovative treatments, warranting further research and collaboration among stakeholders from both high- and low-income nations.

## Plain Language Summary

Advanced liver cancer, also known as hepatocellular carcinoma (HCC), remains a life-threatening condition. In recent years, the introduction of targeted therapies, angiogenesis inhibitors, and immune checkpoint inhibitors has significantly improved clinical outcomes for many patients. However, these advances come at a high cost. The resulting financial burden, often termed financial toxicity, is a major concern, particularly in low- and middle-income countries (LMICs), where access to such treatments is limited. This review examined recent evidence on novel systemic therapies for HCC, barriers to treatment access, and potential strategies to improve affordability. The findings highlight that treatment cost is the primary factor limiting the use of immunotherapy in LMICs. Several practical cost-containment approaches have shown promise, including: Dose rounding and vial sharing to minimize drug wastage, Optimized dosing schedules, such as lower doses or extended dosing intervals, and Shortened treatment durations when supported by efficacy and safety data. Early clinical and pharmacoeconomic studies suggest that these strategies can maintain therapeutic benefit while substantially reducing costs. Broader implementation of such approaches, coupled with coordinated efforts among governments, healthcare systems, and pharmaceutical companies, could help expand global access to life-saving therapies for HCC.

## Graphical abstract

**Graphical Abstract:**
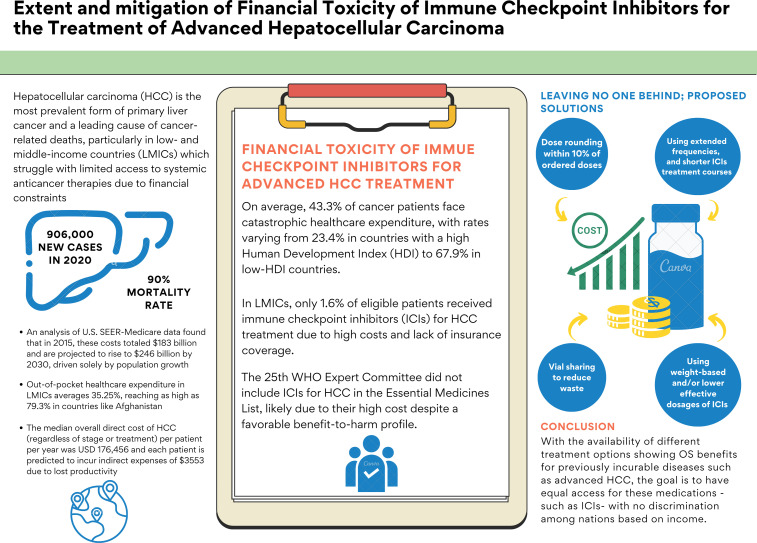

## Highlights

1. The financial toxicity of treating advanced HCC is of major concern, particularly in low- and middle-income countries (LMICs), whereby HCC patients often have very limited access to new treatments, including ICIs.
2. Mitigating financial toxicity with extended frequencies, shorter ICI courses and lower effective doses can facilitate affordable treatment worldwide, including in settings were rising drug prices strain insurance systems.
3. Implementing different strategies like dose rounding and vial sharing can break barriers, expanding ICI access globally.
4. Encouraging further research addresses urgent unmet need, improving wider ICI access across all income settings and healthcare system types.

## Introduction

Primary liver cancer (PLC) ranks as the sixth most prevalent malignancy across the globe and is considered among the top 5 solid tumors with high mortality rate.^1^ In 2020, PLC accounted for 8.2% of all cancer-related deaths, making it the third leading cause of cancer mortality globally.^1^ From 2012 to 2018, the number of new cancer cases in the low- and middle-income countries (LMICs) increased by 1.2 million, with cancer-related deaths increasing from 5.3 million to 6.7 million during the same period.^2,3^ The World Health Organization (WHO) estimated that in 2020 there was globally around 906 000 new cases of PLC with almost 90% mortality rate.^1^ Hepatocellular carcinoma (HCC) is the most prevalent form of PLC and constitute a significant source of mortality and economic burden worldwide, particularly in LMICs.^4^

LMICs face common underlying challenges when it comes to health expenditure. In 2020, as an example, the health expenditure in the LMICs, as a percentage of gross domestic product (GDP), was less than half that in the high-income countries (5.62% vs 14.02%).^5^

This is particularly problematic when it comes to the treatment of HCC, where the LMICs share common difficulties, including poverty and unaffordability, limited access to systemic anticancer therapies, low health literacy and a lack of awareness regarding cancer prevention and treatment, and healthcare infrastructure, and inequity.^6-8^

In a recent global study, 5% of cancer-related disability-adjusted life years (DALYs) are attributed to liver cancer alone.^9^ PLC is most common in Asia and West Africa and for both cancer incidence and mortality in Mongolia, Cambodia, India and Egypt.^1^ Worth mentioning that all these countries belong to LMICs according to the World Bank classification.^5^ In many LMICs, including those in Asia and West Africa, the true burden of PLC is likely underestimated. This is largely due to incomplete cancer registry coverage, limited population-level screening programs, and underdiagnosis, all of which contribute to underreporting and an inaccurate picture of the actual incidence and mortality rates of HCC.^10,11^

In oncology literature, financial toxicity (FT) is defined as both the objective financial burden and subjective financial distress resulting from a cancer diagnosis and its treatment.^12^ Objective FT encompasses measurable economic impacts such as out-of-pocket expenses, medical debt, bankruptcy, and depletion of savings, while subjective FT includes psychological distress, anxiety, and reduced quality of life associated with financial strain. According to the European Society for Medical Oncology (ESMO) expert consensus statements, FT has been implicated in psychosocial, economic, and other harms that lead to suboptimal cancer outcomes across the entire trajectory of diagnosis, treatment, supportive care, survivorship, and palliation. This adverse effect is particularly pronounced in resource-limited settings where social safety nets are minimal.

The FT of treating advanced HCC is troublesome for every nation even those with high-income, but it is particularly concerning in LMICs. In these regions, patients frequently struggle to access even basic healthcare services and affordable essential medications.

FT was first described in 2013 by Zafar et al,^13^ since then the term has been widely used but with collective agreement about its Definition.^14-16^ Recently, cancer excerpts came into consensus about the definition of FT as both the objective financial burden and subjective financial distress from a cancer diagnosis and treatment.^12^

The experts concluded that FT combines the cumulative financial strain that arises from out-of-pocket (OOP) medical expenditure, indirect expenses, and the need for financial adjustments. Moreover, it includes the depletion of personal assets, and the ongoing financial challenges experienced by individuals and their families due to cancer. FT usually start with diagnosis and persist throughout treatment, supportive care, palliative care, and often extends into survivorship.^12^ Even in High income countries, cancer care poses substantial burden on both healthcare systems and patients. A study by Mariotto et al (2020) analyzed cancer-attributable medical care costs in the U.S. using SEER-Medicare data. They found that in 2015, these costs totaled $183 billion and are projected to rise to $246 billion by 2030, driven solely by population growth.^17,18^ Notably, the highest per-patient costs occurred during the end-of-life phase, averaging $105,500 for medical care and $4200 for prescription drugs.^19^

We conducted an exploratory targeted review of the current literature aiming to provide insights into the FT of ICIs use in HCC patients with a focus on LMICs and shed some light on promising proposed solutions to tackle this important problem.

## Treatment Options of Advanced HCC

The choice of advanced HCC treatment depends mainly on performance status, Child-Pugh score, baseline Alpha Fetoprotein (AFP) level and the ability of the patient to afford the cost of these treatments. Although radical removal of the tumor is a potentially curative treatment option for HCC; recurrent disease is extremely common in early-stage disease and no intervention is proven to lower the estimated 60 to 70% recurrence risk of early-stage HCC after procedures with curative intent (such as radical resection and local treatments, including radiofrequency ablation).^20^ Immune checkpoint inhibitors (ICIs) represent a novel approach in the advanced setting. Recently, ample relevant clinical studies have surfaced, particularly related to the use of ICIs and exploring the therapeutic potential of ICI combination strategies.^21,22^

Patients should be encouraged to enroll in clinical trials assessing immune-based combinations or ICI monotherapy as adjuvant treatment, with a view to lower risk of recurrence and improve clinical outcomes in this aggressive disease.^23^ However, in many LMICs, the availability of such trials is very limited, posing an additional barrier to ICIs access. Moreover, the absence of robust trial infrastructure and the geographic concentration of research centers further limit opportunities for eligible patients to participate, leaving many in LMICs at a disadvantage with fewer chances to receive advanced and potentially life-saving treatments.^24^

Since 2008, Sorafenib was the only available treatment for patients with advanced HCC.^25^ However, new options including vascular endothelial growth factor (VEGF) inhibitors, newer tyrosine-kinase inhibitors (TKIs) and ICIs have demonstrated superior efficacy.^26^ IMbrave150 Phase III trial showed superiority of atezolizumab bevacizumab combination over sorafenib monotherapy, and after having attended more than a decade of ‘stagnation', the HCC medical community has a new standard of care.^27^

It is worth mentioning that ICIs monotherapy or immune-based combinations have been tested, but none showing to outperform the standard treatment and not currently used in the standard practice.^28^ First line options include atezolizumab in combination with bevacizumab or lenvatinib monotherapy, sorafenib or most recently durvalumab with or without tremelimumab. While second line options include nivolumab in combination with ipilimumab as opposed to various monotherapy options including regorafenib, ramucirumab, cabozantinib, sorafenib or Lenvatinib if not used in first line.^29^ Pembrolizumab or nivolumab monotherapy can be considered as option only in certain circumstances in the second line settings as category 2B level of evidence in the international guidelines.^29^ Overall, these are medicines that portend a limited benefit to patients, especially in the later lines, and are associated with elevated costs and broad disparities for access.

## Does HCC Survival Correlate with Income?

Despite the fact that survival from PLC remain poor even in high income countries^30^; patients diagnosed with PLC in LMICs are expected to have a worse 5-year survival rate due to disparities in income and access to treatment.^31^

In Uganda (5-y OS: 3.2%) and Gambia (3%) the expected outcome is poorer than reported in Denmark (9%) and USA (14.8%), related to a limited access to therapeutics.^32^ Moreover, low-income and rural-neighborhood patients had advanced HCC tumors and higher mortality.

These disparities likely reflect suboptimal care, especially in HCC surveillance.^33^ Another study concluded that poverty and limited access to healthcare may increase the likelihood of developing HCC and shorten the patients’ OS time.^31^

## Financial Toxicity of Advanced HCC Treatment

The exorbitant cost of cancer treatment may obstacle patients’ access to proper care.^34^ According to a meta-analysis of 19 studies, a significant proportion of cancer patients (43.3% on average) experienced catastrophic healthcare expenditure (CHE).^35^

CHE is defined as the OOP health payments that exceed the financial capacity of the individuals. The extent of such expenditure varied widely depending on the Human Development Index (HDI) of the country where the study was conducted.

In countries with the highest HDI, the percentage of cancer patients who incurred CHE was 23.4%, while in countries with the lowest HDI, this figure was as high as 67.9%.^35^

Various factors were found to be associated with CHE. Among these factors was the type and location of cancer, where HCC resulting in the highest rates of CHE.^36^ Notably, even in high income countries, nearly 85% of cancer patients quit their workforce during initial treatment according to a meta-analysis.^37^ Furthermore, a longitudinal study in United States concluded that nearly 42% of cancer patients lost their entire life savings within the first 2 years of treatment.^38^

In Egypt, a decision analytic model result (after 4 years) of advanced HCC patients highlighted that the total costs for the sorafenib and best supportive care (BSC) cohorts were US dollars 4 229 940 and US dollars 3 092 886 respectively.^39^

Another study in India concluded that the lifetime cost of treating advanced HCC patient using sorafenib and BSC was US dollars 4554 and US dollars 3095 per patient, respectively.^40^

In 2021, the average GDP per capita of LMICs, as reported by the World Bank, was roughly US dollars 2,573, with the countries allocating between 4% and 7% of their GDP to healthcare expenditures.^41^ This allocation varies greatly by country, with some countries dedicating a greater proportion of their GDP to healthcare than others. According to the World Health Organization, Sierra Leone only spent 2.6% of its GDP on healthcare in 2018, whereas Cuba spent 10.6% of its GDP on healthcare.^41^ Given these limited resources, public funding for healthcare is often insufficient to cover high-cost cancer therapies, shifting much of the financial burden onto patients and their families. As a result, even the least expensive treatments may constitute a large amount of an individual’s annual income.

Considering the cost of advanced HCC treatment in the context of GDP and healthcare spending, it is evident that these treatments can represent a significant financial burden for individuals and healthcare systems in LMICs, especially considering that these nations typically have limited healthcare resources. The vast majority of cancer patients in LMICs are unable or unwilling to pay for their cancer care.^42-44^ Moreover, in most LMICs, a public health insurance covering cancer treatments is very limited.^45^

Lack of health insurance, lower income, unemployment and younger cancer diagnosis predicted severe financial burdens.^46,47^ Most advanced HCC patients in LMICs have very limited accessibility to the new treatments including ICIs. In a recent real world study conducted in India, it was found that among all patients who were indicated to received ICIs (9610 patients), only 1.6 % (155 patients) actually receive ICIs.^48^ Moreover, according to the World Bank data, OOP expenditure (% of current health expenditure) in LMICs is 35.25% on average, reaching the highest percentage of 79.30% in Afghanistan, 64% in Cambodia, 63% in Egypt, 38% in Uganda, and 23% in Gambia.^49^ This means that due to the lack of insurance or governmental copayment, the patients must pay for their health care cost out of their own pocket.

When it comes to HCC, the median overall direct cost (regardless of stage or treatment) per patient per a year was US dollars 176 456 and each patient is predicted to incur indirect expenses of US dollars 3553 due to lost productivity.^50^

The average wholesale direct costs of ICIs used in advanced HCC management (in US dollars)^51^ (Table 1) account to: US dollars 11818.37 for atezolizumab, US dollars 13796.7 for durvalumab, US dollars 2821.3 or US dollars 4231.9 or US dollars 8463.88 for nivolumab (different dose ranges available), US dollars 38593.61 for ipilimumab, US dollars 40 000 for tremelimumab and US dollars 12820.22 or US dollars 25640.44 for pembrolizumab (different dose ranges available).

**Table 1.** The Average Wholesale Direct Cost of Immune Checkpoint Inhibitors Used in Advanced Hepatocellular Carcinoma

Drug name	Doses used in HCC	Place of therapy	Available strength	Average wholesale price (US dollars)	Average direct cost per cycle (US dollars)	Remarks
Atezolizumab	1200 mg	1^st^ line	840 mg^a^	8272.86	11818.37	Every 3 weeks in combination with bevacizumab
			1200 mg	11818.37		
Durvalumab	1500 mg	1^st^ line	120 mg^a^	1103.74	13796.7	Every 4 weeks, either in combination with tremelimumab or monotherapy
			500 mg	4598.90		
Tremelimumab	300 mg	1^st^ line	300 mg	300 mg	40 000	Used for a single dose of tremelimumab in combination with durvalumab every 4 weeks
Nivolumab	1 mg/kg	2^nd^ line	40 mg	1410.65	*2821.3	Every 3 weeks in combination with ipilimumab for 4 cycles
			100 mg	3526.61		
	240 mg		240 mg	4231.9	4231.9	Monotherapy every 2 weeks
	480 mg		480 mg	8463.88	8463.88	Monotherapy every 4 weeks
Ipilimumab	3 mg/kg	2^nd^ line	50 mg	9648.43	*38593.61	Every 3 weeks in combination with nivolumab for 4 cycles
			200 mg	38 593.61		
Pembrolizumab	200 mg	2^nd^ or	100 mg	6410.11	12820.22	Every 3 weeks
	400 mg	3^rd^ line			25640.44	Every 6 weeks

^a^This dose is not used for advanced HCC.

*Dose based on average adult weight of 60 kg, NA: details are not available in RED Book. The monthly wholesale costs for individual agents were approximately as of late 2022 data.

In addition to the high direct costs of medications, the FT of HCC in LMICs is strongly shaped by indirect expenses and structural healthcare system challenges. HCC can result in significant productivity losses, with patients experiencing an average of 20 days of absenteeism annually,^52^ and indirect costs accounting for 14-21% of the overall economic burden in some LMICs.^53^

This burden is amplified by the geographic centralization of oncology services in urban centers, forcing patients and families from rural areas to incur substantial travel and lodging expenses, which often lead to treatment delays or abandonment.^54,55^ In India, outstation patients face direct non-medical costs nearly 15 times higher than local patients due to the need to travel to major cities for care.^53^

Healthcare system financing models further influence the degree of FT. In many LMICs, universal health coverage remains limited, and OOP payments account for up to 73% of total healthcare spending.^44,56,57^ Even within existing public healthcare systems, essential cancer medications are frequently excluded from national formularies, compelling patients to turn to private markets where costs are often unaffordable. Patients treated in private or for-profit facilities are therefore at higher risk of CHE compared to those receiving care in public institutions. Moreover, fragmented or mixed healthcare systems contribute to duplication of costs, poor coordination across levels of care, and inconsistent insurance coverage, leaving large segments of the population under-insured or entirely unprotected.^46^ The scarcity of oncology specialists and advanced treatment facilities in many LMICs further exacerbates existing disparities in access to care.^58^

Collectively, the interplay of healthcare financing models, service centralization, and inadequate insurance or social safety nets drives both objective (CHE) and subjective (distress, hardship) dimensions of FT for cancer patients in LMICs.

The expert consultation meeting on cancer medicine candidates for the 2025 Model Lists of Essential Medicines (EML) indicated that, among all cancer medicine applications received, the highest priorities for addition to EML were pembrolizumab (for dMMR/MSI-H colorectal cancer, non-small cell lung cancer (NSCLC) with PD-L1 ≥50%, and cervical cancer CPS ≥1). Also, atezolizumab, and cemiplimab for NSCLC were included. Notably, ICIs for HCC, including atezolizumab + bevacizumab, durvalumab (alone or with tremelimumab) were not supported for inclusion on the EML.

The committee believed that the benefit-to-harm ratio of ICIs was favorable in selected indications, however, listing of ICIs used in HCC was not suggested; most probably due to high costs, and concerns over global affordability.^59^

## Leaving No One Behind; Proposed Solutions

ICIs play a significant role in the treatment of HCC^21,22^; however, they pose huge burden on the healthcare systems and patients due to their high cost. Therefore, Therefore, identifying innovative solutions to improve access to treatments, particularly ICIs, is critically important for patients with advanced HCC in LMICs as summarized in Table 2. Dose rounding was proven to be a successful method for waste and cost reduction in oncology care.^60^ In case of weight-based dosing for nivolumab and ipilimumab; dose rounding within 10% of the ordered doses is expected to substantially reduce the cost and drug waste without impact on medications effectiveness.^60^ Vial sharing is another strategy to reduced waste and improve cost saving particularly in large or publicly funded institutions.^61^

**Table 2.** Proposed Solutions Not to Leave Anyone Behind

Mitigation Strategy	Description	Key evidence/references
Dose rounding	Rounding doses (within 10%) to reduce drug waste and cost without affecting efficacy	Fahrenbruch et al. 2018^62^
Vial sharing	Sharing unused vial portions among patients/institutions to minimize waste	Gilbar et al. 2022; Hall et al. 2020^63,64^
Weight-based dosing	Using body weight to tailor dosage, often reducing cost compared to fixed dosing	Chang et al. 2022; Goldstein et al. 2017; Low et al. 2022^65-67,71^
Lower dosage regimens	Administering lower-than-standard doses that still achieve PD-1 receptor saturation	Jiang et al. 2023^68^
Extended dosing intervals	Increasing time between doses while maintaining efficacy and safety	Cantini et al. 2023; Van den Heuvel et al. 2022^69,74^
Shorter treatment courses	Reducing total duration of ICI treatment for comparable outcomes	Abraham et al. 2022^80^
Off-label/adaptive dosing	Innovative frequency or off-label dosing; dramatic cost reductions, more patients treated	Patel et al. 2022^83^
Policy & system-level	Price negotiation, value-based pricing, procurement, aid/network for leftover meds	Bognar et al. 2016; Ruff et al. 2016; Patel et al. 2022^7,8,83^

Using weight-based dosing strategies instead of fixed dose in combination with vial sharing was also proven to provide cost saving in the case of pembrolizumab and nivolumab, these 2 strategies saved almost 1.5 million US dollars over only 4 months.^70^ A study conducted in Taiwan found that giving pembrolizumab with a dose of 1.8 mg/kg achieved similar OS when compared with standard 200 mg every 3 weeks.^72^ Freshwater et al performed budget impact simulation of pembrolizumab using weight-based dosing (2 mg/kg) compared to fixed 200 mg dose, they found that using the 2 mg/kg dosing produced annual savings of around US dollars 0.825 billion.^73^

Based on pharmacological rationale, a real-world study from Singapore showed that using weight-based dosing of ICIs minimized the cost by almost 13 million US dollars, the cost savings for patients getting pembrolizumab and nivolumab would amount to US dollars 55,692 and US dollars 5335 per patient, respectively.^73^ It is important to note that race and population characteristics can affect optimal dosing strategies, and dosing protocols should be tailored accordingly to ensure both efficacy and cost-effectiveness across diverse patient populations. Outside clinical trial stetting and based on pharmacokinetic rationale, The extended interval dosing in patients with cancer receiving immune checkpoint inhibitors (EDICI) study showed that extended interval dosing of ICIs did not cause any unexpected or new safety signals.^74^

Dosing modifications, such as extending the time between infusions can substantially reduce medication and administration costs, while maintaining clinical effectiveness and a favorable safety profile. Such strategies are particularly valuable in resource-limited settings, as they may expand access to treatment for a greater number of patients without increasing the risk of adverse outcomes.^75^

There is evidence suggesting that ICIs can achieve considerable activity at doses far below those currently approved.^76^ These evidence are supported by preclinical data suggesting that programmed death-1 (PD-1) receptor occupancy was achieved at very low doses; 90% of PD-1 receptors was saturated at 0.5 mg/kg pembrolizumab, 3 mg/kg avelumab, 4 mg/kg atezolizumab and totally saturated at 0.3 mg/kg durvalumab and 0.3 mg/kg nivolumab.^62,63,64,77^ Moreover, the trough concentration (Cmin) using approved dose (1200 mg) of atezolizumab is 20 times higher than the Cmin achieved using 3 mg/kg in the preclinical trial.^62,65,66^

Using a fixed lower nivolumab dose of 20 mg or 100 mg every 3 weeks compared to standard 3 mg/kg biweekly; Yoo et al showed that among 47 NSCLC patients using low dose along with extended frequency achieved better objective response rate than standard approved dose and frequency.^67^ Similar results using 100 mg or 140 mg nivolumab were proven by Zhao et al in renal cell carcinoma patients.^69^

In a phase 2 trial of melanoma patients using fixed low dose 10 mg of adjuvant nivolumab for 1 year was shown to be an efficient and financially advantageous alternative compared to conventional dosing.^78^

Pembrolizumab was also tested in 114 advanced NSCLC patients using low fixed dose of 100 mg and showed no differences in OS or PFS compared to standard pembrolizumab dosing.^68^ In a case control simulation study done by the Veterans Health Administration, the authors concluded that adopting weight-based dosing, dose rounding, and vial sharing could reduce annual ICIs spending by $74 million (13.7%).^79^

Not only can ICIs be effective when used in lower doses but also when used with extended frequencies and for shorter time than approved legalization. Real world data from a LMIC confirmed that shorter course (median of 3 months only) of ICIs achieved comparable safety and efficacy as conventional treatment regimen.^80^ The preliminary data of the NVALT-30 clinical trial showed no significant OS difference between low pembrolizumab dose (100 mg every 3 weeks or 300 mg every 6 weeks) and standard dose.^81^ In a recent study, Karlsson et al evaluated optimized dosing strategies for PD-1 and PD-L1 inhibitors, including dose reduction, extended intervals, and vial sharing, through pharmacokinetic simulations. These approaches achieved cost reductions of up to 69% and carbon emission decreases of 63% while maintaining therapeutic exposure, demonstrating their potential to promote sustainable and cost-effective oncology care.^75^

The preliminary results from the phase II randomized, open-label PLANeT trial, presented at the ESMO 2025 Congress, demonstrated that a one-tenth dose of pembrolizumab (50 mg every 6 weeks) achieved a pathologic complete response rate comparable to the standard regimen (200 mg every 3 weeks) in patients with early-stage triple-negative breast cancer.^82^

Taking all these considerations together, A. Patel et al proposed off-label dosing and frequencies for pembrolizumab (1 mg/kg every 6 weeks), nivolumab (0.6 mg/kg every 4 weeks), and atezolizumab (2 mg/kg every 6 weeks). With this strategy, cost reductions of 86%, 93%, and 95% were achieved, enabling treatment for 7, 14, and 21 additional patients, respectively.^83^

International collaboration is important to tackle this challenge. Such partnerships could be established through joint funding initiatives involving high-income and LMIC institutions, the development of regional cancer research networks, and global alliances (such as WHO-led programs or international oncology societies) that promote technical assistance and knowledge sharing.

LMICs may also benefit from adopting care coordination models similar to the “patient follows doctor” approach used in some high-income countries, which can improve continuity of care and treatment adherence.

Additionally, implementing health information-sharing networks across institutions and regions can streamline cancer care delivery, reduce redundant procedures and testing, and ultimately decrease the overall cost of HCC management. Also, collaborative trial consortia and capacity-building efforts can help incorporate LMIC centres into global research initiatives, enhancing access to innovative therapies and reducing financial disparities.^84,85^

Pharmaceutical companies should actively participate in these cost-reduction strategies through investigating these strategies in clinical trials and promoting such practice in their educational campaigns.

Such measures may help decrease the FT associated with advanced HCC. Large studies describing the consequences of using decreased doses and extended intervals on the cost of therapy, especially in LMIC, and how they would affect the room for innovation are required in future research.

## Policy and System-Level Recommendations

Improving access to ICIs requires not only clinical innovation but also policy-level engagement. National drug price negotiations, value-based pricing models, and reimbursement reforms have gained traction globally as mechanisms to curb the escalating cost of oncology care. In LMICs, these efforts may involve partnerships with international organizations, licensing agreements, and pooled procurement strategies. However, even in high-income settings, payer restrictions and formulary limitations necessitate system-wide approaches to enhance affordability.

To effectively reduce FT, it is important to implement not only pricing and reimbursement reforms but also broader systemic changes. In many LMICs, cancer services are often concentrated in major urban hospitals, requiring patients to travel long distances and face significant non-medical expenses. Strengthening regional cancer centres and extending services beyond capital cities can help alleviate these disparities. Also, investing in the oncology workforce through training and retaining oncologists, oncology nurses, and pharmacists, can enhance local access and minimize the hidden costs associated with delays in diagnosis and treatment.

Moreover, supportive policies are necessary to alleviate the indirect costs that patients and families incur, such as subsidized transportation, housing near treatment facilities, and caregiver support programs.

Collaborative research platforms that generate local pharmacoeconomic data, coupled with regulatory openness to adaptive dosing schedules, could create scalable frameworks for equitable ICI access.

## Limitations

LMICs share common features, a low GDP per capita according to the world bank data and that OOP expenditure represents a substantial portion of total health expenditure in these countries which makes such high-cost cancer treatments burdensome.

Moreover, it will reflect a similar FT among their HCC patients.

In our manuscript, we have tried to highlight the different challenges faced by LMICs, but we acknowledge that a more thorough analysis of the literature is necessary to describe the current situations in each different country and region. An additional limitation would be that the costs of advanced HCC treatments can vary greatly between countries due to sales agreements and pricing negotiations with pharmaceutical companies. Our intention in presenting these prices in Table 1 is to provide a general idea of the cost range for these treatments. The prices presented in Table 1 are meant to be illustrative and not definitive, as they may vary depending on the country and pricing agreements. Moreover, the indirect cost may be hard to capture, especially unemployment is widespread, and people are not involved in economic activities. Another limitation is that the analysis may primarily focus on direct treatment costs, overlooking the broader economic impact on societies. Also, many studies may focus on short-term outcomes, failing to capture long-term financial impacts of ICIs. Most of the studies exploring the reduced doses of ICIs with or without the extended frequencies were tested in the real-world setting across different malignancies; but further research is needed to conclude a solid reproduceable conclusion.

Moreover, there is a paucity of economic evidence supporting these strategies specifically in HCC, with current data primarily extrapolated from studies in other malignancies. Consequently, the application of these cost-saving approaches in HCC requires judicious clinical decision-making by the medical team, alongside careful consideration of regulatory and medico-legal frameworks.

The clinical evidence supporting low-dose and extended-interval ICI regimens in HCC consists primarily of retrospective observations. Most published data derive from non-HCC malignancies (NSCLC, melanoma, RCC) with different tumor immunobiology.

No randomized controlled trial comparing low-dose vs standard-dose regimens has been completed in HCC. These strategies remain entirely off label as no pharmaceutical manufacturer has sought regulatory approval for modified dosing regimens.

## Conclusion

An outstanding matter to address; with the availability of different treatment options showing OS benefits for previously incurable diseases such as advanced HCC, the goal is to have equal access for these medications -such as ICIs-with no discrimination among nations based on income. While LMICs face disproportionate challenges in affording ICIs, the economic strain is also increasingly felt in wealthier nations grappling with unsustainable drug prices. Several proposed initiatives may potentially improve access to ICIs if validated through HCC-specific research. These include dose rounding, vial sharing, lower dosage, extended frequencies and shorter ICIs treatment courses. Implementation should occur within research protocols until prospective trials demonstrate non-inferiority of efficacy. These must be complemented by systemic changes in pricing and reimbursement, across all income settings. Moreover, another initiative to improve access to ICIs would be establishing of a network or a society that deals with management of collecting of left-over drugs. Further research -with primary focus of HCC- should be encouraged to address this unmet need, allowing more patients in LMICs to gain access to these vital treatments.
